# Hashtag fitspiration: credibility screening and content analysis of Instagram fitness accounts

**DOI:** 10.1186/s12889-023-15232-7

**Published:** 2023-03-02

**Authors:** Rachel G Curtis, Ivanka Prichard, Georgia Gosse, Anna Stankevicius, Carol A Maher

**Affiliations:** 1 Alliance for Research in Exercise, Nutrition and Activity, UniSA Allied Health and Human Performance, University of South Australia, GPO Box 2471, Adelaide, South Australia 5001 Australia; 2 College of Nursing and Health Sciences, SHAPE Research Centre, Flinders University, GPO Box 2100, Adelaide, South Australia 5001 Australia

**Keywords:** Instagram, Fitspiration, Social media, Content analysis, Credibility

## Abstract

**Background:**

Fitspiration is a social media phenomenon purported to inspire viewers to lead healthier lifestyles but can result in negative psychological outcomes such as body dissatisfaction. This study aimed to develop a tool to audit Instagram fitspiration accounts and screen for content that could have potentially negative psychological effects.

**Methods:**

This study developed and implemented an audit tool to (1) identify credible fitspiration accounts (i.e., accounts that do not portray potentially harmful or unhealthy content) and (2) describe the content of identified accounts. The most recent 15 posts of 100 leading Instagram fitspiration accounts were audited. Accounts were deemed non-credible and were excluded if they contained fewer than four fitness-related posts or portrayed nudity or inappropriate clothing, sexualisation or objectification, extreme body types, “thinspiration”, or negative messages.

**Results:**

Many accounts contained fewer than four fitness-related posts (n = 41), sexualisation or objectification (n = 26), nudity or inappropriate clothing (n = 22), and/or extreme body types (n = 15). Three accounts failed on all four criteria, while 13, 10 and 33 failed on three, two, or one criterion, respectively. Therefore, only 41% of accounts were considered credible. Inter-rater reliability (percentage agreement and Brennan and Prediger’s coefficient κ_q_) was high (Stage 1: 92% agreement [95% CI 87, 97], κ_q_ 0.84 [95% CI 0.73, 0.95]; Stage 2: 93% agreement [95% CI 83, 100], κ_q_ 0.85 [95% CI 0.67, 1.00]). Account holders of credible fitspiration accounts were predominantly female (59%), aged 25–34 (54%), Caucasian (62%), and from the United States (79%). Half held a qualification related to physical activity or physical health (e.g., personal trainer, physiotherapy; 54%). Most included accounts included an exercise video (93%) and example workout (76%).

**Conclusion:**

While many popular Instagram fitspiration accounts offered credible content such as example workouts, many accounts contained sexualisation, objectification or promotion of unhealthy or unrealistic body shapes. The audit tool could be used by Instagram users to ensure the accounts they follow do not portray potentially harmful or unhealthy content. Future research could use the audit tool to identify credible fitspiration accounts and examine whether exposure to these accounts positively influences physical activity.

**Supplementary Information:**

The online version contains supplementary material available at 10.1186/s12889-023-15232-7.

## Background

Insufficient physical activity is major public health concern, heightening the risk of non-communicable diseases and negatively impacting mental health and quality of life [1]. Mass-reaching public health campaigns to promote physical activity are needed. Online social networks offer the ability to reach vast numbers of people and are increasingly being used to deliver public health interventions and messaging. Facebook was the most popular platform in early approaches [2], with recent studies using other social networks (e.g., Pinterest, Twitter, WhatsApp) [3]. These researcher-led interventions draw on behaviour change theory and techniques and have been evaluated in small-to-moderately sized samples, but have not been translated to mass-reaching, ongoing campaigns [2, 3].

Instagram is one of the most popular online social networks, with 1.3 billion users globally in 2021 [4]. It is ranked as the third favourite social media platform across all ages (the favourite among females aged 16–34) [4], and shows high engagement compared with other platforms (i.e., 2–7% of users interact with each post on Instagram compared to 0.1–1.5% on Facebook) [5]. Fitspiration Instagram accounts (e.g., health and fitness influencers) share photos and videos of exercise and healthy eating and inspiring quotes to empower individuals to engage in healthy lifestyle choices [6]. Popular fitness inspiration hashtags on Instagram such as #fitspiration and #fitspo currently return over 100 million posts.

The majority of fitspiration posts consist of images of thin and athletic women promoting exercise, fitness, and healthy lifestyles. On face value, this type of inspiration should increase exercise behaviour and help to improve wellbeing. However, a relatively small but growing body of evidence has examined the short-term effects of Instagram fitness content on exercise inspiration, mood, and body image, and suggests that this may not be the case. For example, pre-post between-person experimental studies comparing the effects of viewing different types of imagery (e.g., fitspiration and travel inspiration) have established that, while exposure to fitspiration images inspires fitness, it also results in greater body dissatisfaction and negative mood [7–9] and lower perceived sexual attractiveness [10]. Longitudinal experimental research showed that daily exposure to fitspiration images for 28 days was associated with higher growth in negative mood and appearance comparison compared to neutral or body-positive images, though some growth in positive mood was also observed [11]. Additionally, a cross-sectional survey examining women’s usual behaviour on Instagram found that more frequent viewing of fitspiration content was associated with greater thin-ideal internalization and disordered eating symptomatology [12]. Further, the potential harms from fitspiration have also been outlined in content analyses [6, 13, 14]. These studies suggest that the limited variety of body shapes on display (with the most prominent being an ultra-fit and thin physique) implies that only thin and toned bodies are considered healthy and beautiful. This, along with a strong focus on the appearance and objectification of the body rather than body functionality, promotes appearance-based reasons for exercise, which are known to be associated with increased body image concern and disordered eating symptomatology [15]. However, some types of fitspiration content may be helpful for inspiring exercise intentions. For example, exposure to functionality-focused imagery of women’s bodies actively moving (e.g., #ThisGirlCan) has been shown to increase state appearance satisfaction and exercise intentions [16]. In addition, this type of imagery is considered more inspirational and achievable than non-functionality focused imagery (e.g., selfies) [17]. These findings demonstrate the importance of examining content on social media that negatively or positively impact upon individual body image, exercise behaviour, and subsequent wellbeing.

Few studies have examined whether fitness content on Instagram impacts physical activity behaviour. Two experimental studies found no change in distance travelled on a treadmill following exposure to fitspiration imagery relative to control imagery [8, 9]. However, a pilot study of a home-based Instagram-delivered exercise program reported trends towards improvements in physical activity in young women (n = 9) [18]. An alternative approach may be to harness existing, popular fitspiration accounts to benefit population health. Indeed, followers of fitspiration hashtags report being inspired to exercise and eat healthily [19]. However, anecdotal evidence suggests that the quality and nature of content varies between fitspiration accounts. For such accounts to be used in public health approaches, credible accounts (i.e., accounts that do not portray potentially harmful or unhealthy content) must be identified. This study set out to begin to address this under-researched area. Specifically, we aimed to describe the content and credibility of leading Instagram Fitspiration accounts. In doing so, we describe the development and test-retest reliability of a purpose-designed audit tool. If credible accounts can be identified, they may present an avenue for wide-reaching, engaging public health campaigns to promote physical activity.

## Methods

This study is an audit of the profile and posts of publicly viewable Instagram fitspiration accounts. Ethics approval was provided by the University of South Australia Human Research Ethics Committee (protocol P060-10).

### Sample selection and data collection

A Google search was conducted using the search phrase “top Instagram fitspiration accounts” on Sept 16, 2019. The first 50 search results (individual webpages) were examined, and all fitspiration accounts mentioned were recorded (294 accounts with 432 total mentions). Fifty webpages were considered sufficient to capture relevant fitspiration accounts as the number of new fitspiration accounts diminished as the search continued, until no new accounts were mentioned. Instagram accounts mentioned on more than one webpage were included in the audit, as they were assumed to be popular pages receiving attention outside of Instagram (n = 60). Accounts mentioned a single time (n = 234) were then ordered according to number of followers. The 40 accounts with the highest number of followers were selected for inclusion in the audit (total n = 100). A video recording was taken of each account holder’s bio (a small section under the account holder’s username where they can describe themselves and their Instagram account) and their most recent 15 posts (Sept 24–26, 2019). Fifteen posts were chosen as this is the number of posts that are visible in the initial grid format of Instagram that are viewable when first clicking on an account.

### Data analysis

A standardized audit tool was developed to screen each Instagram account to identify credible accounts, and to describe the account holder and content of credible accounts, based on the account holder’s bio and most recent 15 posts. The tool comprised a two-stage screening process and content analysis. Screening criteria were based on literature examining the effects of viewing certain types of imagery and messaging, as described below. All Instagram accounts were audited by two independent raters (GG, AS) using the final version of the tool (Additional file 1). Disagreements at both stages were resolved by a third independent rater.

Stage 1 screening required the rater to assess the images or videos of the most recent 15 posts. This rapid screening stage was completed by examining the Instagram feed without opening individual posts. Based on extant literature and previous content analyses [e.g., 6, 13, 14], images that depicted nudity or inappropriate clothing, sexualisation or objectification, and extreme body types were a primary concern. Additionally, the authors determined that four fitness-related posts out of the most recent 15 were required to ensure fitness-related content would be regularly viewable by users, while allowing accounts to also include other types of content. To conduct stage 1 screening, raters viewed the Instagram feed of each account and assessed whether the most recent 15 posts contained images or videos depicting (1) nudity or inappropriate clothing (i.e., lingerie or bikini in a non-workout context such as modelling), (2) sexualisation (i.e., sexual posing such as an arching back) or objectification (i.e., images focused on one specific body part such as breasts, buttocks, or muscular abdominals), (3) extreme body types (i.e., extreme thinness or muscularity), or (4) fewer than four (out of 15) fitness-related posts (i.e., images or videos of an individual actively engaging in physical activity). Items were marked “yes” if the content was present, and the account was excluded if any items were marked with “yes”.

Stage 2 screening required the rater to open the 15 most recent posts to further examine the images and their captions and hashtags. Given the potential negative impact of image manipulation [20] as well as demonstrated negative impact of exposure to ‘thinspiration’ [21], posts were assessed on whether they contained (1) suspected image manipulation (i.e., altered body shape evident by blurred, disproportionate or unnatural lines or shapes; filters that changed the lighting of the image were not considered image manipulation), (2) promotion of “thinspiration” ideals (i.e., content that promotes excessive weight loss or disordered eating behaviours, statements with negative connotations to being overweight, or thin praise), (3) dysfunctional quotations (i.e., quotations encouraging unhealthy or excessive attitudes towards the body, diet, or exercise), or (4) “thinspo” hashtags (e.g., #thinspo, #thinspiration). Items were marked “yes” if the content was present, and the account was excluded if any items were marked with “yes”.

Content analysis was performed on accounts that passed both stages of screening and described prevalence of different types of content (e.g., example workouts, exercise videos, hashtags, healthy eating posts), characteristics of the account holder (e.g., gender, age, ethnicity, relevant qualifications related to physical activity or physical health, published programs), and whether the account had a verification badge (blue tick indicating that Instagram has verified the account’s authenticity).

An initial version of the tool was piloted on a subset of accounts, leading to three changes to the stage 1 screening criteria. Firstly, accounts could be inadvertently excluded for depicting a person in minimal clothing in a context where this would not be considered inappropriate. Criterion 1 was therefore updated to provide examples of when minimal clothing is considered appropriate (during physical activity) and inappropriate (when the primary focus of the image is on the body rather than the activity). Additionally, it was determined that raters would make more consistent judgements for criteria 1, 2, and 3 with the assistance of visual examples, therefore example images were added. Finally, criterion 4 originally required five fitness-related posts among the most recent 15 posts, however this led to the exclusion of several accounts that provided quality example workouts and did not fail any other criteria. The requirement was therefore lowered to a minimum of four fitness-related posts. Minor changes were made to the wording throughout for clarity and brevity (see Additional file 1 for the final version of the audit tool).

Inter-rater reliability was based on percentage agreement and Brennan and Prediger’s agreement coefficient κ_q_ [22], undertaken using Stata 17 (StataCorp LLC, College Station, TX). Descriptive statistics (n and %) were used to describe the prevalence of characteristics of the Instagram accounts.

## Results

### Screening

One hundred accounts were screened at stage 1, with 41 accounts proceeding to stage 2. Of the n = 59 excluded accounts, n = 41 were excluded for having fewer than four fitness-related posts, n = 26 accounts for sexualisation or objectification, n = 22 accounts for nudity or inappropriate clothing, and n = 15 for extreme body types. Three accounts failed on all four criteria, while 13, 10 and 33 failed on three, two, or one criterion, respectively. No accounts were excluded at stage 2. Inter-rater reliability was excellent for inclusion/exclusion at stage 1 (n = 100; 92% agreement [95% CI 87, 97]; κ_q_ 0.84 [95% CI 0.73, 0.95]) and stage 2 (n = 41; 93% agreement [95% CI 83, 100], κ_q_ 0.85 [95% CI 0.67, 1.00]).

### Content analysis

**Fig. 1:**
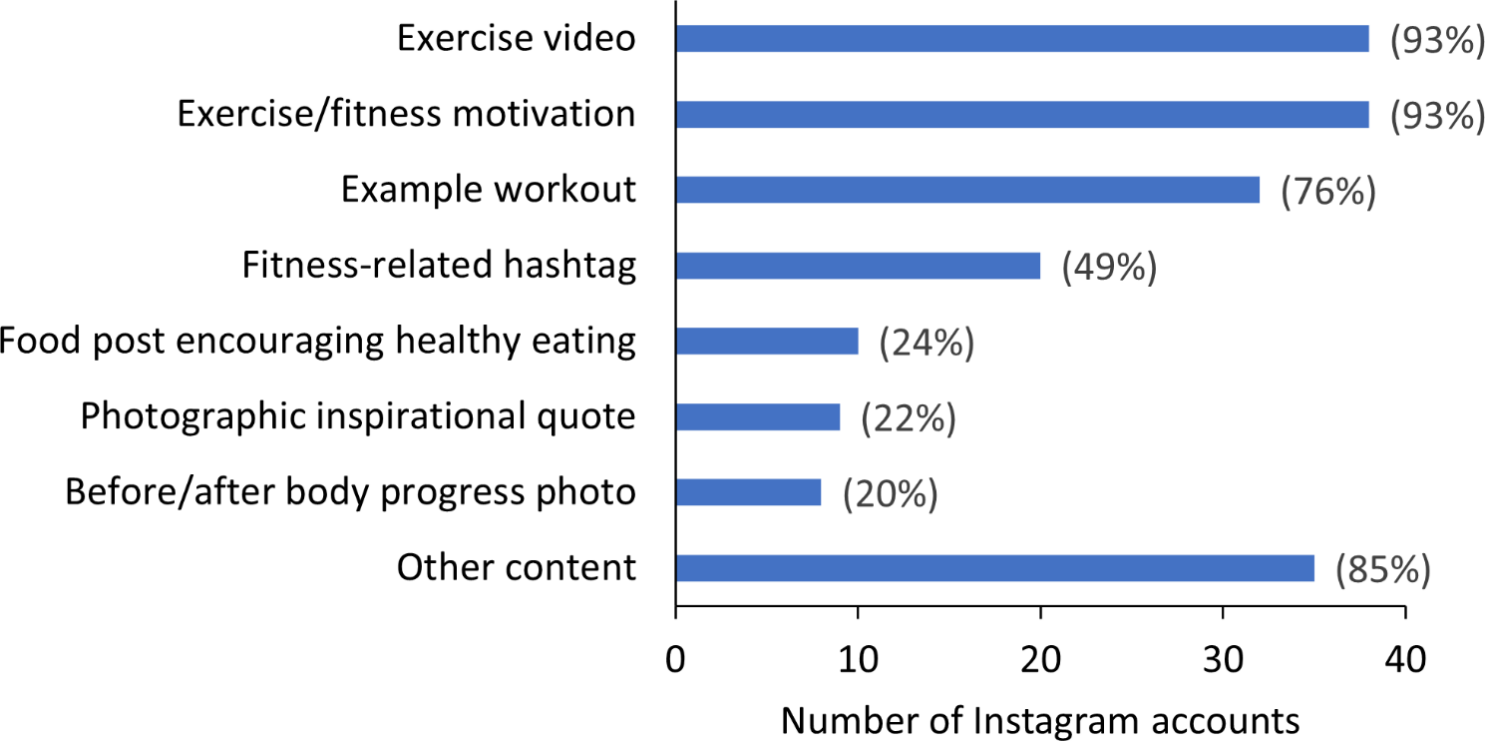
Prevalence of different types of content in included Instagram accounts (n = 41)

Figure 1 summarises the prevalence of different types of content in the 41 included Instagram accounts. Most accounts included an exercise video (n = 33), example workout (n = 32), fitness-related hashtag (n = 20), and targeted exercise motivation [(n = 38) e.g., improving strength (n = 8), improving appearance (n = 4), feeling good (n = 4), reaching specific goals (n = 2; powerlifting 400lbs, completing a marathon)]. Approximately one in five accounts included photographic inspirational quotes (n = 9) and body progress photos (n = 8). One quarter included food posts (n = 10). Most accounts also included other content (n = 35), for example photos of the account holder with family members (n = 9), alone (n = 3) or with pets (n = 2), advertisements (n = 4), posts about clothing and fashion (n = 3), health advice (n = 3), and scenic photos (n = 3).

Table 1 shows characteristics of the account holders of included Instagram accounts (n = 39 because two accounts identified no primary account holder). Account holders were predominantly female, aged 25–34, Caucasian, and from the United States. Half held a relevant qualification and half were an ambassador for a commercial product or brand. The most promoted products were supplements/sports drinks (n = 8), sports clothing/active wear (n = 7), and cars (n = 2). Eight account holders stated these affiliations in their Instagram bio, whereas 11 promoted products in posts only. Half of account holders had published a commercial workout program, such as a smartphone app.

**Table 1:** Characteristics of account holders of included Instagram accounts (n = 39)

Characteristic	n	(%)
Female	23	(59)
Age		
18–24	1	(2)
25–34	21	(54)
35–44	16	(41)
45	1	(3)
Country		
United States	31	(79)
Australia	4	(10)
Other	4	(10)
Ethnicity		
Caucasian	24	(62)
Hispanic	7	(18)
African American	5	(13)
Asian	3	(8)
Qualification (Yes)^a^	21	(54)
Personal trainer	15	(38)
University degree	5	(13)
Other	5	(13)
Brand ambassador (Yes)	19	(49)
Published exercise program (Yes)	21	(54)
App	12	(31)
Website	5	(13)
Book	4	(10)
Instagram verification	36	(92)

^a^Categories do not add up to 21 as some account holders had multiple qualifications

## Discussion

This study describes the development and application of an audit tool for identifying and describing Instagram fitspiration accounts that do not portray potentially harmful or unhealthy content. The tool was easy to use and had excellent inter-rater reliability. 59% of the fitspiration Instagrammers failed to meet our threshold for credibility and were omitted after the first stage of screening. Of the fitspiration Instagrammers deemed credible based on the screening tool, the majority were female, from the US, and Caucasian. Just over half of them had a health-related qualification, typically at a diploma level. We note that all account exclusions occurred at stage 1 screening. It may therefore be appropriate to conduct this rapid screening stage without the stage 2 screening in some instances, though future research should confirm this finding.

Of concern, nearly two-thirds of the popular accounts were deemed to be not credible. Many of these contained hyper-sexualisation or promotion of unhealthy or unrealistic body shapes. Previous content analyses have also revealed high levels of sexualization and objectification of body parts such as the abdomen and buttocks in fitspiration images [6, 23] with images of women more likely to contain sexualization and objectification than images of men [23, 24]. Research suggests that, while the proportion of fitness-focused images posted by female fitness influencers has increased between 2019 and 2021, levels of sexualization and objectification have remained the same [24]. Given the links between exposure to this type of imagery and mood and body image concern [7–12], the proportion of accounts containing such imagery is alarming. Individual Instagram users may benefit from education to understand the potential negative effects of following non-credible fitspiration accounts and could use the audit tool, which is freely available with this paper, to identify potentially harmful imagery and inform their decision of whether to follow an account. Future research should examine how viewing credible fitspiration content influences body dissatisfaction and mood.

Conversely, results suggested that many popular Instagram fitspiration accounts do offer credible content, such as example workouts, without including content likely to result in body dissatisfaction or other negative psychological effects. Following these accounts could conceivably increase motivation and exercise education, leading to improvements in heath. While research found that exercise exertion was not greater immediately after viewing fitspiration content [8, 9], exposure to screened credible content, as well as exposure over a longer period, might show different effects. Initial pilot work suggests a 12-week home-based Instagram-delivered exercise program can improve in physical activity in young women [18]. Future experimental research is now needed to examine the effects of exposure to different types of fitspiration content on exercise intention, behaviour, and wellbeing. If positive effects of credible fitspiration content are found, individuals interested in improving their physical activity could use the current audit tool to aid their selection of credible accounts to follow.

The research findings and audit tool may also be useful for public health initiatives. Social media platforms including Instagram impact social and economic outcomes [25]. Within the commercial marketing arena, social media influencer marketing is recognized as a legitimate avenue for driving brand-recognition and sales, representing a $10 billion industry in 2020 [26, 27]. In contrast, public health promoters have been slower to harness this modality, though examples of governments working with Instagram influencers are emerging, for example, to encourage social distancing strategies and vaccine uptake during the COVID pandemic [28, 29]. Future work examining opportunities for physical activity promotion on Instagram appear warranted. This might include identifying credible accounts to partner with, developing new credible accounts using the audit tool as an initial framework, or providing training for social media influencers to ensure their accounts are credible. Research is needed to determine whether exposure to such social media content can translate into measurable health behaviour change.

Fitspiration account holders that were deemed credible after screening were typically young Caucasian females from the United States. These attributes do not contribute to account credibility and are likely to reflect the platform user-base (e.g., a previous content analysis found women appeared in social media fitspiration images more frequently than men [6]), or a potential bias in the webpages used to identify fitspiration accounts. However, a lack of representation of diverse populations should be considered if Instagram fitspiration accounts are to be used for public health promotion. In addition, research suggests that greater diversity in body shape and size is needed to promote positive body image on social media [e.g., 30]. As such, researchers could usefully audit social media account holders across different popular hashtags (e.g., #bodypositive).

### Strengths and limitations

A key strength of the current study was its novelty. This is the first published study to attempt to develop an evidence-based audit tool for Instagram fitspiration accounts. The tool has excellent inter-rater reliability and face validity, and is provided in full in this paper’s appendices, making it freely available for use or adaptation (e.g., where it could be applied to other forms of content such as body positivity, body functionality, or to fitspiration material on other platforms such as #FitTok on TikTok). Applying the audit tool to 100 popular fitspiration accounts at the time of the audit provides an overview of the quality and content of these accounts. It may serve as a baseline to which future quality and content audits may be compared.

Limitations must also be acknowledged. Being a new tool in a new field, there are limited avenues for validating the tool, beyond face validity. Furthermore, by design, Instagrammers are continually creating new content. Like any audit tool, this tool is applied in a limited timeframe, and it is possible that accounts deemed “credible” when audited, may not be at a different time (and vice versa). Finally, the content analysis was completed using data available within Instagram; it is possible that some of the account holders had qualifications or commercial programs which were not obvious from their Instagram profiles.

Given the popularity of Instagram, and the high number of people using Instagram to seek health and fitness content, there appears to be considerable opportunities for health promoters to harness this for population health benefit. The audit tool presented in this study helps identify credible vs. non-credible Instagram accounts. Future research is warranted to understand whether exposure to this screened fitness content can positively impact physical activity behavior and health without causing negative psychological effects. To date, most health and fitness related research regarding Instagram has been observational or qualitative in nature, and experimental research designs would help advance this field of research. In addition, the current audit focused on fitness, but observed that many popular fitspiration accounts identified also provide dietary advice. Future research examining the content of diet advice on social media, and potential opportunities for health promotion, would be beneficial.

## Conclusion

This study reports the first audit of popular Instagram fitspiration accounts, including the development of a repeatable tool for auditing such accounts. Around two-thirds of the leading fitspiration accounts audited lacked credibility or contained potentially harmful or unhealthy content. However, the remaining third of accounts appeared credible. Given the natural appeal and large following of Instagram fitness personalities, opportunities for harnessing credible Instagrammers to achieve population physical activity promotion warrant further exploration.

## Electronic supplementary material

Below is the link to the electronic supplementary material.

Supplementary Material 1

## Data Availability

The datasets used and/or analysed during the current study are available from the corresponding author on reasonable request.
