# Supplementary material for: Hashtag fitspiration: credibility screening and content analysis of Instagram fitness accounts
Source: BMC Public Health. 2023 Mar 2;23:421. doi: 10.1186/s12889-023-15232-7 (PMC9979526; doi:10.1186/s12889-023-15232-7)
Supplement: Supplementary file 1 — Supplementary Material 1 [file 12889_2023_15232_MOESM1_ESM.docx]

**SCREENING** Stage 1

Examine the images of the most recent 15 posts and indicate whether the content is present. Can be conducted on the home page without opening posts. Proceed to stage 2 if none are present.

| **Yes**  **⃝** | 1. **Nudity or inappropriate clothing** Inappropriate clothing includes minimal clothing (e.g., bikini, bathers, or lingerie) in a non-workout context (e.g., bikini modelling or tanning on beach) *when the primary focus of the photo is the body* (Image A)*.* Females in workout clothes such as a sports bra, or males shirtless, whilst engaging in physical activity is accepted (Image B). |
| --- | --- |
| ⃝ | 1. **Sexualisation or objectification** Sexualisation includes a luring or sultry gaze, winking, or sexual posing such as an arching back (Image C). Objectification includes images focused on one specific body part such as breasts or buttocks for females and muscular abdominals or biceps for males (Image D). |
| ⃝ | 1. **Individual body shape appears extremely thin, or excessively muscular**   Extremely thin: unnaturally thin with little to no visible fat or muscular stores, prominent bony eminences (Image E). Extremely muscular: body building figure (Image F). |
| ⃝ | 1. **Fewer than 4 posts are fitness-related**   Fitness-related posts are posts that include images/videos of an individual engaging in physical activity/exercise. Record number of fitness-related posts______­­­­­_____ |
|  | ***EXCLUDE account if any items are marked “Yes”*** |

**SCREENING** Stage 2

Open and examine each of the most recent 15 posts (image/video, caption, and hashtags) and indicate whether the content is present. Proceed to content analysis if none are present

| **Yes**  **⃝** | 1. **Suspected image manipulation**   Altered body shape evident by blurred, disproportionate or unnatural lines or shapes, which may or may not be consistent with other images of the same individual. Filters on Instagram photos are not considered manipulation. |
| --- | --- |
| ⃝ | 1. **Promotion of “thinspiration” ideals**   Images, videos, or captions that promote excessive weight loss or disordered eating behaviours (e.g., skipping meals). Statements with negative connotations to being overweight, and thin praise (e.g., “you look your best when you are your skinniest”)*.* |
| ⃝ | 1. **Dysfunctional quotation**   Extreme quotations taken too far encouraging unhealthy or excessive attitudes towards the body, diet, or exercise. |
| ⃝ | 1. **“Thinspo” hashtags**   Captions, comments, or hashtags related to thinspiration e.g., #thinspo, #thinspiration, #skinnygoals |
|  | ***EXCLUDE account if any items are marked “Yes”*** |

**CONTENT ANALYSIS** Description of content

Examine the most recent 15 posts and indicate whether the content is present. Provide descriptions where required.

| **Yes**  **⃝** | 1. **Example workout**   Image or video demonstrating a series of exercises in a workout, with brief instructions that followers can complete themselves. |
| --- | --- |
| ⃝ | 1. **Active exercise video**   Video depicting an individual actively completing exercise(s). May or may not include instructions to complete the exercise(s). |
| ⃝ | 1. **Fitness-related hashtag**   Post tagged with hashtag(s) related to fitness (e.g., #fit, #fitspiration, #fitness, #exercise, #healthy, #workout). |
| ⃝ | 1. **Exercise/fitness motivation**   Example of motivation for exercise or fitness (e.g., appearance, strength, mental health, competition).  *Provide description of motivation(s)*____________________________________________ |
| ⃝ | 1. **Photographic inspirational quote**   An inspirational quote/phrase in large text formatted as an image (e.g., an individual post, not a caption). Inspirational quote to have a positive message regarding personal improvement, goals, physical activity/exercise, fitness, or a healthy lifestyle. |
| ⃝ | 1. **Before/after body progress photo**   Photo of self or others comparing the body before and at a defined period after commencing a personal fitness journey. |
| ⃝ | 1. **Food post encourage healthy eating**   Image, video, or text related to food or nutrition that promotes healthy eating (e.g., photo of breakfast, meal preparation, recipe ideas). Do not tick “yes” if post(s) contain: 1) food restraint not due to a medical condition, 2) guilt associated with food consumption, or 3) encouragement to replace foods with supplements. |
| ⃝ | 1. **Other content**   Other content not included in the above categories.  *Provide description of other content* _________________________________________________ |

**CONTENT ANALYSIS** Description of account holder

Examine the account holder’s bio and most recent 15 posts. Answer the multiple-choice items and provide descriptions where required.

|  | Gender | ⃝ Male | ⃝ Female | ⃝ Other |  |
| --- | --- | --- | --- | --- | --- |
|  | **Age** | **⃝** 18- 24 | **⃝** 25-34 | **⃝** 35-44 | **⃝** 45+ |
|  | *If not listed, age is approximated based on images of the account holder* | | | | |
|  | **Country** | Please specify_______­­­­_____________ **⃝** Not stated | | | |
|  | **Ethnicity** | ⃝ White/Caucasian | ⃝ Indian | ⃝ Asian | ⃝ Indigenous |
|  |  | ⃝ African American | ⃝ Hispanic | ⃝ Other/Not sure |  |
|  |  |  |  | *If “other”, please specify____________* | |
|  | **Instagram Verification**  Account verified by the Instagram Verification tick? | | | ⃝ Yes | ⃝ No |
|  | **Qualifications**  Does the individual have any relevant qualifications (e.g., Certificate III Fitness, Personal Trainer, Physiotherapy, Exercise Physiologist)? | | | ⃝ Yes | ⃝ No |
|  |  |  |  | *If “yes”, please specify_______________* | |
|  | **Brand ambassador**  Is the individual an ambassador for a particular brand (e.g., food or clothing), organisation, or charity? | | | ⃝ Yes | ⃝ No |
|  |  |  |  | *If “yes”, please specify______________* | |
|  |  |  |  | *If “yes”, is this identifiable in the bio?* | |
|  |  |  |  | ⃝ Yes | ⃝ No |
|  | **Published physical activity/exercise program**  Has the individual created published, sellable physical activity content for purchase (e.g., as book/e-book/smartphone app/website)? | | | ⃝ Yes | ⃝ No |
|  |  |  |  | *If “yes”, please specify_______________* | |
|  |  |  |  | *If “yes”, is this identifiable in the bio?* | |
|  |  |  |  | ⃝ Yes | ⃝ No |

Images for **SCREENING** Stage 1

*All images are from pexels.com and are freely available for use without attribution*


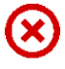

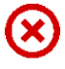

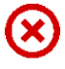

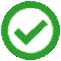

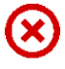

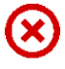


Extremely muscular

Objectification

Extremely thin

Minimal clothing, image focussed on the body

Bikini while exercising at the beach

Sexualisation
